# Abdominal diffuse dermal angiomatosis resolved with panniculectomy

**DOI:** 10.1016/j.jdcr.2025.05.055

**Published:** 2025-08-22

**Authors:** Sydney DeVore, Megan Carrigan, Jonhan Ho, Teun Teunis, Joseph C. English

**Affiliations:** a Department of Dermatology, University of Pittsburgh School of Medicine, Pittsburgh, Pennsylvania; b Department of Plastic Surgery, University of Pittsburgh School of Medicine, Pittsburgh, Pennsylvania

**Keywords:** angioproliferation, diffuse dermal angiomatosis, hypoxia, panniculectomy, peripheral vascular disease, smoking

## Introduction

Diffuse dermal angiomatosis (DDA) is a very rare vascular disorder caused by reactive skin angioproliferation. The pathophysiology of the angioproliferation has not been entirely elucidated, but is thought to be either ischemia or inflammation leading to local hypoxia, increased vascular endothelial growth factor, and the formation of excess blood vessels.^1^ The condition is a diagnosis of exclusion that manifests as erythematous to violaceous lesions on the skin, which often include painful ulcers or erosions.^2^ Here, we present a case of DDA presenting on the abdomen of a former female smoker with aortic stenosis and peripheral vascular disease.

## Case reports

**Fig 1:**
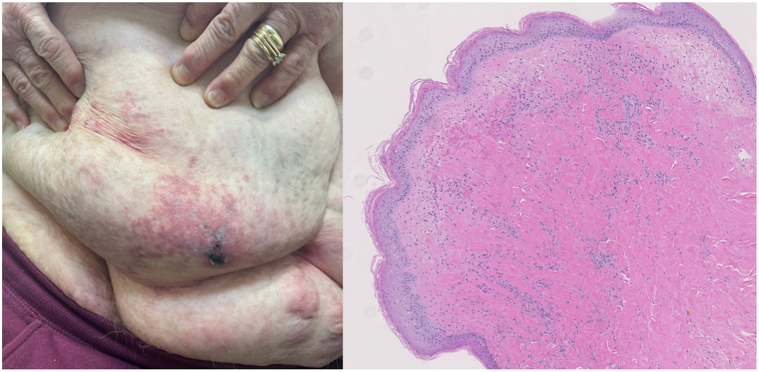
Diffuse dermal angiomatosis initial presentation and dermatopathology (left, ×4).

A 71-year-old female patient presented to the clinic with 1 month of diffuse lower abdominal pannus pain. On examination, the pannus skin demonstrated tender but not indurated reticulated erythema and painful ulcerations with firm induration of the ulcer edge (Fig 1). She had been previously treated in the outpatient setting 3 different times with antibiotics for cellulitis of the abdominal wall. She was eventually admitted inpatient for treatment with intraveneous Rocephin, which led to mild improvement in redness but persistence of the lesions.

The patient’s past medical history included obesity, atrial fibrillation, moderate to severe aortic stenosis, psoriatic arthritis, factor V Leiden, lupus anticoagulant, deep vein thrombosis, and peripheral vascular disease. Her medications included amlodipine, warfarin, hydroxychloroquine, and methotrexate. She is also a former smoker with a 26-pack-year history.

A punch biopsy of the nonulcerated skin (Fig 1) was obtained and revealed dermal thickening, an increase in collagen bundle thickness, and increased interconnecting capillary vascular channels lined by endothelial cells. CD34 stain additionally highlighted increased vascular channels. These findings, in conjunction with the patient’s symptoms, lead us to suspect a diagnosis of DDA.

Peripheral vascular disease workup consisted of a contrast-enhanced abdominal computerized tomography scan demonstrating atherosclerosis of the distal aorta and iliac vessels, but adequate flow into the deep epigastric vessels, and a transthoracic echocardiogram demonstrating severe aortic stenosis. Hypercoagulation workup confirmed that the patient has factor V Leiden and the lupus anticoagulant. Her complete blood cell count, comprehensive metabolic panel, and serum protein electrophoresis were within normal limits.

**Fig 2:**
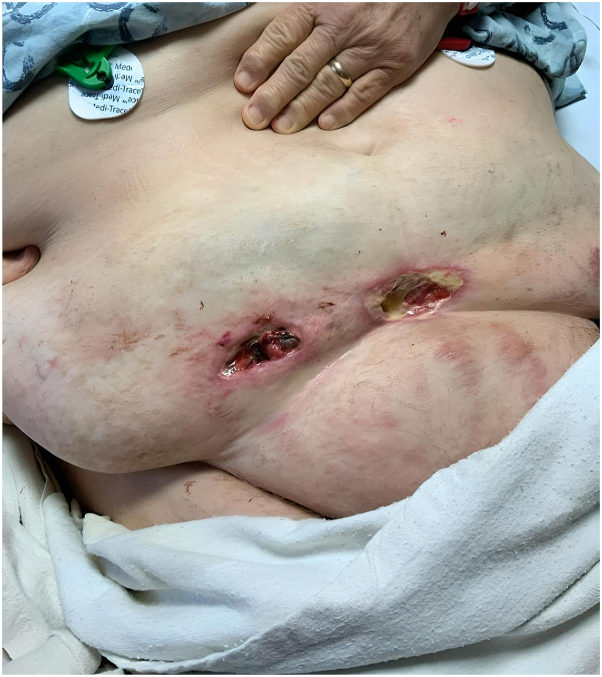
Preoperative diffuse dermal angiomatosis (note: ulcerations worsened).

**Fig 3:**
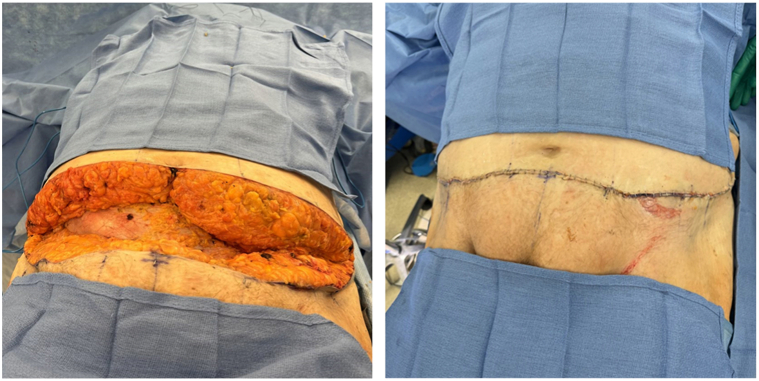
Operative and postoperative panniculectomy for diffuse dermal angiomatosis.

Dermatology recommended weight loss, smoking cessation, and referred the patient to hematology for anticoagulation medication change, vascular surgery for revascularization, and plastic surgery for panniculectomy. Hematology changed warfarin to apixaban for anticoagulation, but it did not improve the pain. Due to the relatively minor atherosclerosis, and adequate flow into the deep epigastric vessels (which are perforating vessels that perfuse the abdominal pannus), vascular surgery deferred angioplasty. The cosmetic dermatology team was consulted to inquire whether liposuction or laser therapy would be suitable treatment options; however, due to a lack of literature to support these options, this was deferred. To address the mass effect of the low-hanging abdominal pannus and worsening ulcerations (Fig 2) as a potential cause, plastic surgery offered panniculectomy (Fig 3). Besides some minor wound dehiscence that healed, the patient recovered well from panniculectomy and has been pain-free without recurrence.

## Discussion

Patients with DDA present with this disease between 40 and 60 years old, with the average age being 54 years.^3^ There is a female gender predominance, with 74% of patients with DDA being women.^3^

DDA is associated with smoking and cardiovascular risk factors such as atherosclerosis and peripheral vascular disease.^4^ A systematic review of 73 reported DDA cases by Touloei in 2019 found that smoking was present in 58% of patients, obesity in 33%, and peripheral vascular disease in 23%. Another systematic review in 2020 found that DDA was associated with diabetes, chronic kidney disease, and hyperlipidemia.^3^ Diseases, such as Takayasu arteritis, chronic obstructive pulmonary disease, calciphylaxis, and hepatitis, have also been previously cited in association with DDA.

The majority of cited DDA cases are present in the breasts and are associated with mass effect of the heavy breast tissue or perhaps vascular insufficiency due to subclavian occlusion.^3^ The lower extremities have also been cited previously, but to our knowledge, fewer than 10 cases of DDA have been reported presenting in the abdomen. The differential diagnosis includes calciphylaxis, livedo vasculopathy, intravascular B-cell lymphoma, patch/plaque stage Kaposi sarcoma, and inflammatory breast cancer.^5^^,^^6^

Treatments for DDA are generally targeted at reversing the cause of tissue hypoxemia. The most common causes of hypoxemia are mass effect of body tissue (such as pendulant breasts or abdominal tissue) or atherosclerosis.^3^ Lifestyle modifications such as smoking cessation and weight loss have been shown to be very effective. Isotretinoin, steroids, and topical tacrolimus/pimecrolimus have been cited previously as effective therapies. However, review of the literature indicates that isotretinoin mainly decreases symptoms of DDA without addressing the underlying pathophysiology. Mass removal of large breasts or pannus removal surgery is generally considered the only curative (salvage) treatment.^7^ In several cited breast DDA cases, mastectomy was performed as a curative therapy when mass effect of the tissue was suspected to be the cause of the tissue hypoxia, and ulceration did not improve with nonsurgical treatments such as isotretinoin. In 1 review of 22 breast DDA cases, mastectomy was more likely than other treatments, such as isotretinoin, to prevent recurrence.^8^ Isotretinoin is often used in breast DDA because of its antiangiogenic role, but this review found that its use was primarily able to decrease symptom severity rather than address the cause of the disease.^8^ Little information is available in the literature about the frequency of panniculectomy as a salvage treatment for abdominal cases of DDA. However, using a similar concept of decreasing tissue burden, it seems to be a reasonable alternative. If major vessel occlusion is suspected as the cause of tissue hypoxia, angioplasty or vascular bypass might be attempted first, or concomittantly.^3^

## Conflicts of interest

None disclosed.
